# Orchard Mapping with Deep Learning Semantic Segmentation

**DOI:** 10.3390/s21113813

**Published:** 2021-05-31

**Authors:** Athanasios Anagnostis, Aristotelis C. Tagarakis, Dimitrios Kateris, Vasileios Moysiadis, Claus Grøn Sørensen, Simon Pearson, Dionysis Bochtis

**Affiliations:** 1 Institute for Bio-Economy and Agri-Technology (iBO), Centre for Research and Technology–Hellas (CERTH), GR57001 Thessaloniki, Greece; a.anagnostis@certh.gr (A.A.); a.tagarakis@certh.gr (A.C.T.); v.moisiadis@certh.gr (V.M.); d.bochtis@certh.gr (D.B.); 2 Department of Computer Science & Telecommunications, University of Thessaly, GR35131 Lamia, Greece; 3 Department of Electrical and Computer Engineering, Aarhus University, DK-8000 Aarhus C, Denmark; claus.soerensen@ece.au.dk; 4 Lincoln Institute for Agri-Food Technology (LIAT), University of Lincoln, Lincoln LN6 7TS, UK; spearson@lincoln.ac.uk; 5 farmB Digital Agriculture P.C., Doiranis 17, GR54639 Thessaloniki, Greece

**Keywords:** precision agriculture, orchard mapping, deep learning, computer vision, semantic segmentation, orthomosaic

## Abstract

This study aimed to propose an approach for orchard trees segmentation using aerial images based on a deep learning convolutional neural network variant, namely the U-net network. The purpose was the automated detection and localization of the canopy of orchard trees under various conditions (i.e., different seasons, different tree ages, different levels of weed coverage). The implemented dataset was composed of images from three different walnut orchards. The achieved variability of the dataset resulted in obtaining images that fell under seven different use cases. The best-trained model achieved 91%, 90%, and 87% accuracy for training, validation, and testing, respectively. The trained model was also tested on never-before-seen orthomosaic images or orchards based on two methods (oversampling and undersampling) in order to tackle issues with out-of-the-field boundary transparent pixels from the image. Even though the training dataset did not contain orthomosaic images, it achieved performance levels that reached up to 99%, demonstrating the robustness of the proposed approach.

## 1. Introduction

The latest advances in sensing technologies dedicated to agricultural systems have led to the emergence and development of a modern management concept, namely precision agriculture, which focuses on efficient management of the temporal and spatial variability of field and crop properties using information and communication technology (ICT) [1]. A plethora of different sensors and technologies are utilized in relation to this concept to form a detailed view of fields’ properties, capturing the spatial and temporal variability and searching for the specific factors responsible for their occurrence, which are to be treated accordingly. Therefore, mapping the field and crop properties is a fundamental aspect in the application of such management systems.

Remote sensing is defined as the non-contact measurement of crop properties based on the radiation reflected from the plants, using ground based or aerial platforms, and it is widely used for mapping tasks in agricultural systems [2]. Recent technological advances have made unmanned aerial systems (UASs), i.e., sensing systems mounted on unmanned aerial vehicles (UAVs), commercially available. These systems provide high spatial resolution images and, in combination with their ease of use, quick acquisition times, and low operational cost, they have become particularly popular for monitoring agricultural fields [3]. Several studies have utilized UASs for crop management purposes, such as yield prediction and site-specific fertilization [4] by capturing multispectral images, irrigation using thermal imaging [5], or for field scouting using RGB (Red-Green-Blue) orthomosaics [6].

In tandem with the development of remote sensing and image capturing techniques, machine learning (ML) has leaped forward both in terms of performance as well as in terms of its applicability in almost all scientific domains. Agriculture in general, and specifically precision agriculture, has benefited from the rise of machine learning in multiple ways since complex tasks, hard to deal with using traditional programming, can be tackled with the help of a plethora of different ML algorithms [7,8,9]. Particularly with regard to computer vision, there has been extensive implementation of machine and deep learning in tasks of classification [10], object detection [11], and semantic segmentation [12].

Classification tasks employ self-learning algorithms for the assignment of a class to an image according to its content. Examples of classification in agricultural applications include the identification of diseases on leaves in real-life environment images with the help of convolutional neural networks (CNNs) [13], the identification of trees from UAV images with a combination of k-nearest neighbors (kNNs) and GoogLeNet [14], and tree species assignment from UAV images and multispectral data with random forest algorithms [15]. Object detection algorithms differ because they predict the location of objects and assign the appropriate class to them [16]. Examples of object detection applications include the detection of disease-infected leaves at tree level from UGV images [17], the detection of trees with the use of Faster RCNN (Regions with CNN features) and YOLO (You Only Look Once) [6], and the mapping of operational environments in orchards with classical computer vision techniques or Fast RCNN [18]. Image segmentation, on the other hand, is a pixel-wise operation where a class is appointed to each individual pixel, thus creating detailed outlines of objects and maintaining their exact shape. U-net [19] is one the most famous algorithms for image segmentation and has been used for tree segmentation from satellite images [20], mapping of forests [21], and pomegranate canopy segmentation in comparison to Mask RCNN [22]. Segmentation algorithms for tree canopy mapping have also been used in tandem with object detection approaches, like Segnet and YOLO [23], or classification approaches, like the multi-resolution segmentation algorithm used with state-of-the-art CNNs and support vector machines (SVMs) [24].

Applications of image segmentation with images acquired by UAS have used several machine learning algorithms: point-cloud data with the use of deep neural networks (DNNs) for tree canopy segmentation [25], support vector machines and image pre-processing filters for citrus trees segmentation [26], random forest (RF) super pixel classification for tree canopy extraction [27], and for the automatic segmentation of canopies with Deeplab v3+, a type of encoder-decoder network, for automatic segmentation of canopies [28].

Several approaches, listed in the bibliography, have attempted to find solutions to the problem of automatic segmentation of trees from aerial images. However, all approaches had as a prerequisite a full, healthy canopy. All aforementioned studies also tackled the problem with methods of unsupervised learning or object detection. These methods present shortcomings, either regarding the identification performance per se or the precise shape of the canopy excluding surroundings. Subsequent tasks, such as canopy size estimation and orchard mapping, rely on the results of these methods; therefore, the respective shortcomings propagate to them as well. According to our knowledge, semantic segmentation has not been implemented in the task of canopy detection of orchards with the use of UAS-derived images. Furthermore, a gap has been identified in the proper identification of tree canopies within orchards, throughout all seasons and in every step of their growth.

This study aimed to propose, develop, and validate an approach for orchard trees segmentation using aerial images based on a custom variant of a deep learning convolutional neural network, namely the U-net network. The purpose was the automated detection and localization of the canopy of orchard trees under various conditions (i.e., different seasons, different tree ages, different levels of weed coverage). The results of this study contribute to the farming community by providing a robust tool for instant scouting of orchard environments by automatically segmenting the tree canopies from aerial images. This work is a preliminary step in the development of an integrated tool to support farmers in decision making.

## 2. Methodology

The proposed approach is structured around data-driven algorithms and computer vision techniques. An annotated dataset was generated from a large number of UAV captured images by masking the canopies of the trees in order to create a large dataset for supervised learning. This annotated dataset was used to train the model with the selected deep-learning algorithm so that it could properly identify tree canopies and segment them from the background. A mask image is produced as an output, containing the shapes of all predicted tree canopies. Following the segmentation, the weighted average of each mask, i.e., its moment, is used for the calculation of its centroid, so that it can be used as a reasonable approximation of the location of the tree’s trunk. Provided that the geodetic coordinates of the photographed location are retained in the orthomosaic images, the tree trunk locations can be computed with high accuracy.

In order to deal with the complexity of orchard environments, in terms of the presence of weeds in the image background, and the high variability in the phenomenology of canopies due to seasonality, a deep learning algorithm, namely U-net, was considered and tweaked to fit the problem’s requirements.

### 2.1. U-Net Variant

**Figure 1:**
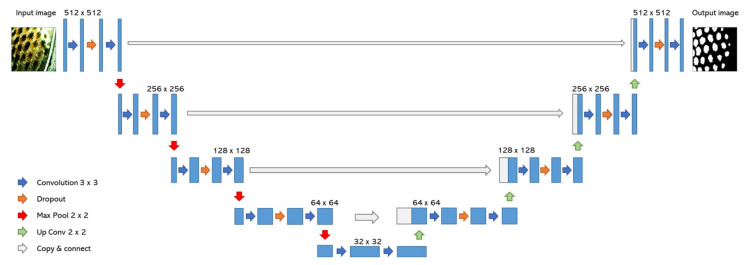
Architecture of the modified U-net network implemented in the approach.

U-net is an advanced type of convolutional neural network which consists of two modules, an encoder and a decoder. Such networks aim to encode the input into a latent space in order to create the desired output based on the said input. U-net’s characteristic feature, which distinguishes it from the case of simple encoder-decoder networks, is that it contains direct “skip” connections between the shallow encoder and decoder layers alongside the sequential structure of the architecture [29]. In this way, certain features from the encoding/input layers are fed directly to the decoding/output layers. For the approach presented here, two modifications were made to the standard U-net architecture; the input layer was tweaked to both 3- and 6-channel images and a dropout layer was added between the convolutional layers per block, to avoid the tendency towards overfitting that would occur in such a small dataset with similar representations. A schematic of the U-net used in this work is shown in Figure 1.

### 2.2. Process Flow

The methodology developed for creating segmentation predictions follows a sequential process which consists of several preprocessing, training, and evaluation steps. The complete process flow can be summarized as follows:

- Data are imported and split into train and test sets. For the implementation of the approach, the test set is required to contain at least one image from each use case;
- Each image is reshaped (into a predefined aspect ratio) and, additionally, color enhancements such as contrast equalization are applied;
- The training data are fed into the U-net and the model learns to create proper segmentations for each image. An evaluation metric is used across a randomly selected validation set comprising 10% of the training set, so that the trained model can learn to create better segmentation masks;
- The trained model produces segmentation masks for the test images and the evaluation metric is applied;
- The segmentation masks are compared with the real masks created during annotation and the presence of false positive or false negative segmentations is manually investigated;
- The overall performance of the model is defined by the accuracy of the trained model on the test dataset, as well as the ratio of false positives and false negatives over the total amount of trees in the image.

**Figure 2:**
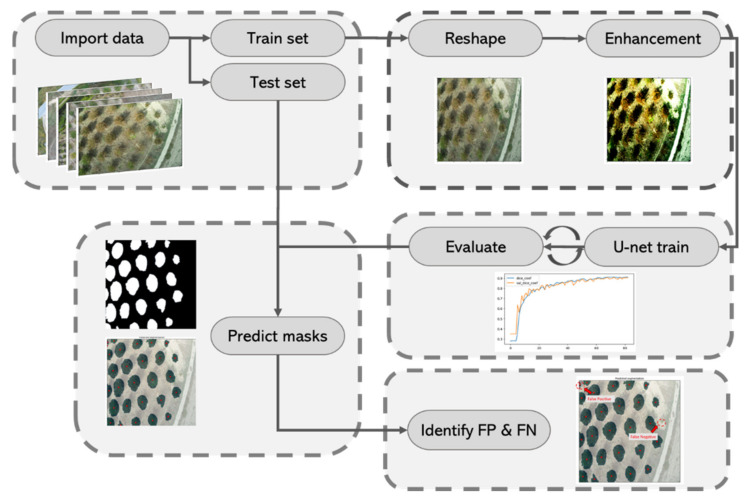
Process flow of the proposed methodology for creating segmentation predictions. FN: false negative; FP: false positive.

A visual representation is shown in Figure 2.

## 3. Implementation

Three sites of commercial walnut orchards, located in Thessaly, Central Greece, were used in the present study. The orchards covered a range of tree ages and soil surface features. Correspondingly, the images represented different seasons, with the aim of capturing the different tree conditions and stages throughout the growing season, namely: defoliated, canopy developing, canopy fully developed, and brown canopy before defoliation. Additionally, the orchards varied in terms of background soil conditions, including: soil surface free from weeds, soil surface partly covered by weeds, and untreated soil with complete weeds coverage. A total number of 106 images from the three orchards led to the definition of seven different use cases which were used for the training and testing of the proposed methodology. A detailed list of the characteristics’ use cases is presented in Table 1.

All use cases were adequately represented by several images in the training set and, more importantly, the test set was constructed so that it would always contain at least one image of each use case. This way, the trained models would be tested for all different combinations of characteristics, ensuring the maximum generalization. Sample images for each use case are presented in Appendix A.

### 3.1. Data Acquisition

From 2018 to 2020, a number of test flights were conducted over the aforementioned orchards with two different types of UAV, a quadcopter (Phantom 4, DJI Technology Co., Ltd., Shenzhen, China) and a fixed-wing UAV (eBee, senseFly, Cheseaux-sur-Lausanne, Switzerland), both equipped with high-accuracy GNSS (real-time kinematic (RTK) positioning) and high-resolution cameras, i.e., 5472 × 3648, at a 3:2 aspect ratio.

The use of RTK GNSS was a requisite to accurately geotag the acquired images. To achieve further exploration, the automated flights were maintained with the necessary criteria to produce high-accuracy orthomosaics. The parameters of each automated flight were fine-tuned (UAV flight height, speed, number of captured images, side overlap and forward overlap ratio) to produce high-resolution, below-centimeter pixel size, orthomosaics, which are presented in detail in Table 2.

### 3.2. Data Pre-Processing

Image pre-processing is a fundamental aspect of computer vision tasks, especially when employing self-learning algorithms. The reason for this is the need to transform the images into proper sizes/shapes, in order for the numerical computations to take place. In the present study, each of the raw images captured from the study sites utilized over 30 MB of storage each and had a 5472 × 3648 pixel rectangular shape. Size reduction was the first step that took place along with the reshaping of all images to dimensions of 512 × 512 pixels.

The effect of image preprocessing in terms of color and colorspaces was another aspect investigated in this study. Histogram equalization (EQ) [30] and contrast-limited adaptive histogram equalization (CLAHE) [31] are two methods usually used for contrast enhancement in RGB images, both of which expand the contrast by adapting the range of the image’s pixel values either globally or locally. Besides the RGB spectrum, the HSV colorspace—which represents color with hue, saturation, and value, all assigned to cylindrical coordinates—was also investigated since it amplifies different features of an image, which could lead to increased performance.

**Figure 3:**
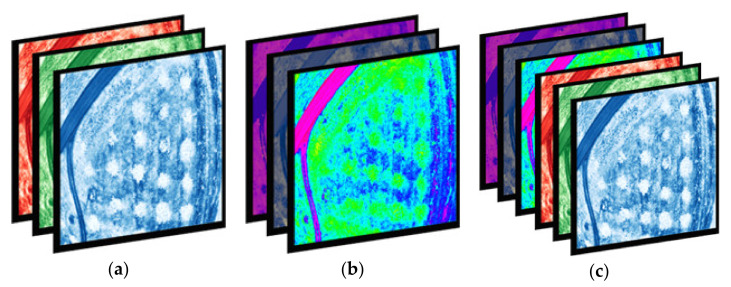
Channel deconstructing of (**a**) RGB, (**b**) HSV, and (**c**) fused images.

A novel approach to increasing contrast and extracting features by combining an RGB contrast-enhanced instance of an image and an HSV instance of an image into a single 6-channel image was attempted. Such images contain “double” information from a regular 3-channel image; however, the addition of more color channels does not necessarily imply that the added value increases as well [32]. A visual representation of how the 3- and 6-channel images are constructed is shown in Figure 3.

**Figure 4:**
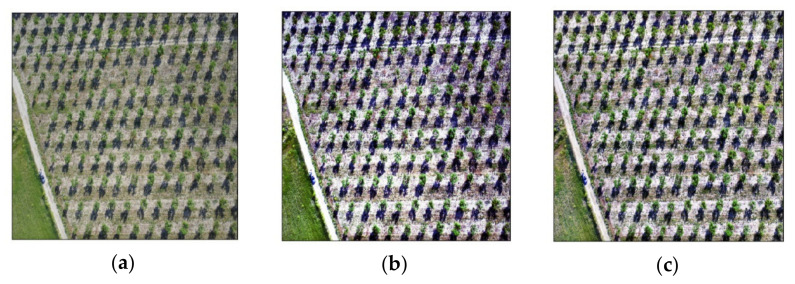

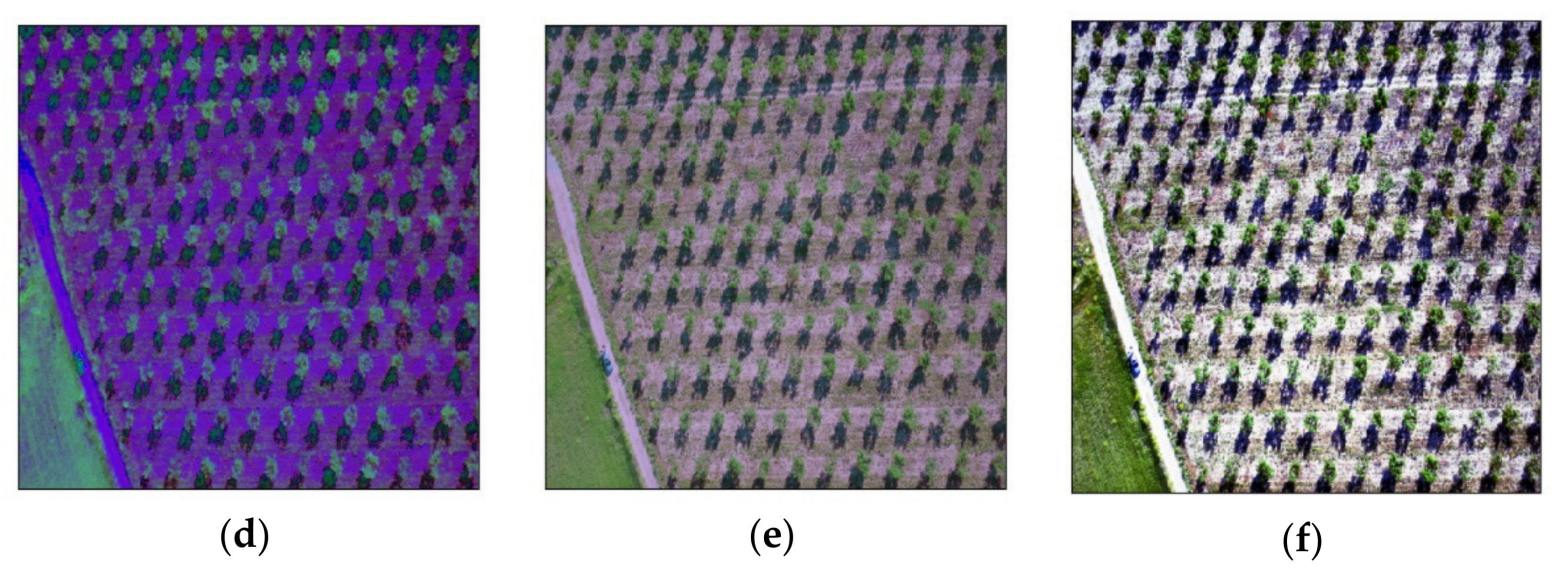
Image color transformations used in the study: (**a**) RGB image, (**b**) EQ image, (**c**) CLAHE image, (**d**) HSV colorspace image, (**e**) 6-channel RGB and HSV fused image, and (**f**) 6-channel CLAHE and HSV fused image.

Two variants of the fused images were tested, namely the RGB image without any contrast enhancement and the CLAHE method for adaptive contrast enhancement, alongside the HSV colorspace image. The visual differences between all methods are presented in Figure 4.

### 3.3. Performance Metric

The Sørensen–Dice coefficient [33] was selected as the performance metric for the segmentation of trees against their background. It was preferred over the intersection over union (IoU, also known as the Jaccard index [34]) because the IoU penalizes bad classifications harder [35] and, in the case of tree foliage, the exact details of the foliage shape is not of high importance. As a loss function, the negative value of the dice coefficient was used, as is common in image segmentation tasks [36].

## 4. Results

### 4.1. Validation on Dataset

**Figure 5:**
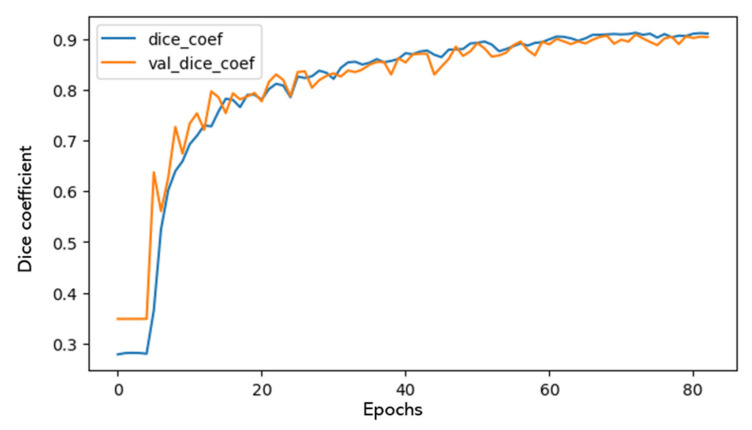
Learning plot with training and validation accuracy.

All models were trained on an NVIDIA Titan 1080 Ti GPU with between 40 and 100 epochs, a visualization of which is seen in Figure 5. Early stopping was used for preventing overfitting of the models. The models were trained and tested on 96 and 10 images respectively, which were randomly selected from the 106 images of the dataset, including all seven use cases (use cases presented in Table 1). In this way, the generalization of the model was ensured. The accuracy achieved by the models under the differently pre-processed datasets is shown in Table 3.

**Figure 6:**
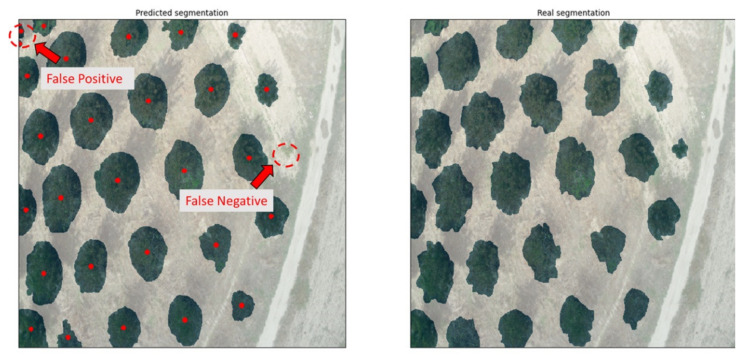
Examples of false positive and false negative segmentation predicted by the developed system (**left**) as compared to the real segmentation (**right**).

As mentioned previously, the dice coefficient is used for benchmarking the performance of trained models. However, the ability of a trained model to properly segment trees is measured by visual inspection. The system was validated by applying the trained models to never-before-seen images of entirely different use cases and comparing the results to the identification of a human expert. The false positives (FPs), i.e., incorrectly identifying trees at locations where there were none, and false negatives (FNs), i.e., failing to identify trees, could thus be registered. On top of the tree canopy segmentation, the exact location of a tree’s trunk was computed based on the predicted masks. The method for computing this location was based on the centroids of the image moments, i.e., the weighted average of the predicted masks. Therefore, for each mask representing a tree canopy, and with the condition that it was isolated and in no way connected to an adjacent mask, a single point was calculated to signify the position of the tree trunk, considering a fairly symmetrical canopy shape. A visual example of the predicted segmentation (left) and the real annotation (right), both overlaid on the original images, is given in Figure 6.

Since this study aimed to primarily solve the issue of mapping the locations of trees within orchards, the absolute intersection between all pixels was mostly considered for the training phase. The rough shape and size of a properly identified tree canopy was what would lead to a correct computation of the trunk location and the estimation of the tree’s age. Therefore, in order to choose the best-trained model for the application, the test set was manually investigated across the predicted segmentations from each approach. In this way, FPs and FNs were identified and, finally, each model received a score based on the ratio of FPs, FNs, and their sum over the total amount of trees in each image, as seen in Table 4.

From the overall evaluation of the models’ performance, the RGB model was identified as the simplest and provided the best results. Therefore, it was selected as the primary model to be investigated further. In the next step, the performance of the RGB model was investigated for each use case separately. In this way, the strengths and weaknesses of the selected approach could be identified and therefore tackled in future work. The results of the RGB method were further broken down per test image, covering all use cases that were included in the present study, as shown in Table 5.

The accuracy achieved for all use cases using the RGB model ranged between 72.7% and 97.9%, which can be considered as a satisfactory result. Comparing images 6 and 7 with 9, the effect of the presence of weeds’ on the accuracy of the model is evident, since the first two images, which belong to use case 6 (large trees; few weeds), performed considerably better compared to image 9, which belongs to use case 7 (large trees; many weeds). In the latter, the FPs were the primary reason for limiting the system’s performance. This signifies that the developed weeds within the image frame led to increased FP misclassifications (weeds classified as trees). Interestingly, when running test images from use cases 1 and 2 (i.e., images captured during autumn when the canopy was turning brown), accuracy was notably high, albeit with a low level of weeds coverage.

**Figure 7:**
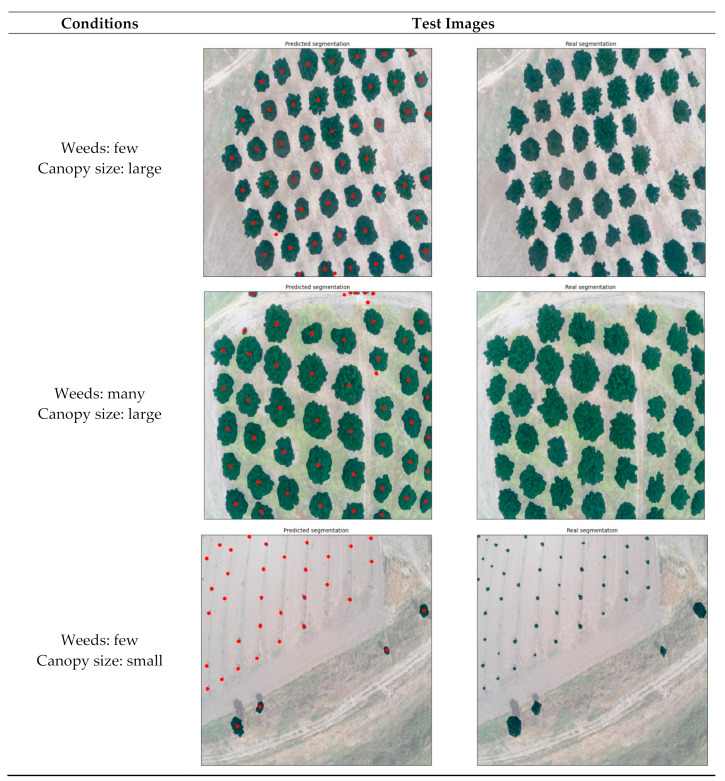
Results of indicative RGB images covering a range of different conditions.

With regard to common characteristics between use cases, three indicative results from the RGB model are presented in Figure 7. These three categories cover the most contrasting situations; (a) ideal conditions with medium/large tree canopies and ground with only a small amount of weeds, (b) intermediate conditions with large tree canopies but weed-infested ground, and (c) unfavorable conditions with small tree canopies and some weeds present. The first image belongs to use case 4, containing clear green canopies and ground covered by only a few weeds. The second image, which represents use case 7, shows large green canopies; however, the ground is almost entirely covered with weeds of a similar shade of green. The third image is from an orchard free of weeds (use case 3); however, the canopies are particularly small in size due to the young age of the trees. Use cases 4 and 6 are the most ideal, considering canopy and background color contrast due to the season and the lack of weeds. A noteworthy outcome is that even though use cases 4 and 5 both had medium-sized canopies, the trained model’s accuracy was completely different due to the presence of weeds. Additionally, use cases 1 and 2 demonstrated similar behavior as use cases 3 and 4, since all of them were almost free of weeds, with the only difference being the more brownish color, making it slightly harder to identify all canopies. In all images, a mask overlay of 50% transparency was applied in order to visualize the segmentations; therefore, the real shades of the images were altered.

### 4.2. Validation on Orthomosaics

The system as presented above showed its ability to recognize tree canopies with high accuracy when applied to high resolution images of certain dimensions. However, investigating the performance of the system with orthomosaics covering the entirety or a large part of the orchard area was also considered to be of great interest. Therefore, in a further analysis, the trained models were applied to orthomosaics captured from orchards with pixel resolution considerably lower than the original training dataset. The aim of this test was to examine the extent of the trained models’ capabilities considering the pixel resolution range of all canopies. Applying the models directly to the orthomosaics produced errors due to the presence of “transparent” pixels that denote areas outside the bounds of the appointed orchard. Two methods were used to overcome this inconvenience: “oversampling”, i.e., filling the transparent pixels with the dominant ground color; or “undersampling”, i.e., cropping the largest area possible that did not contain “out-of-borders” areas.

The test included (a) analysis of orthomosaics treated as a whole (i.e., as one image) and (b) analysis of sub-images clipped from the orthomosaic. It is important to note that these were never-before-seen images that had not been a part of the original dataset. Similarly to the training phase, orthomosaics of three different use cases were selected.

**Figure 8:**
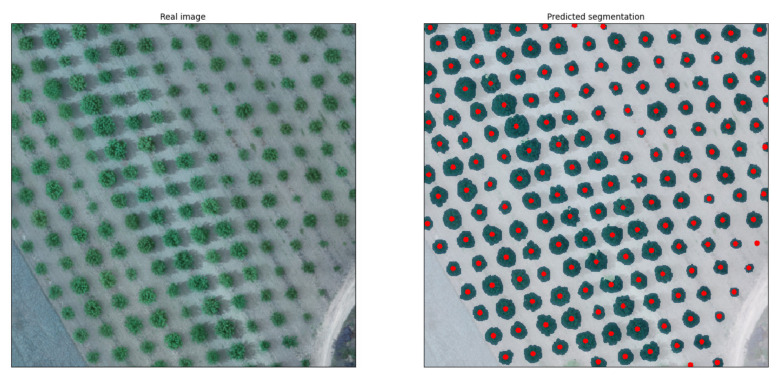
Undersampled orthomosaic of an orchard with large- to medium-sized canopies (**left**) and the segmentation predicted by the model (**right**).

Case A. The first case displayed an orchard with large- to medium-sized canopies. As mentioned above, the pixel resolution was smaller than that of the training dataset. The accuracy reached 99%, with only a small FP segmentation on the right section of the middle of the image detected (Figure 8).

**Figure 9:**
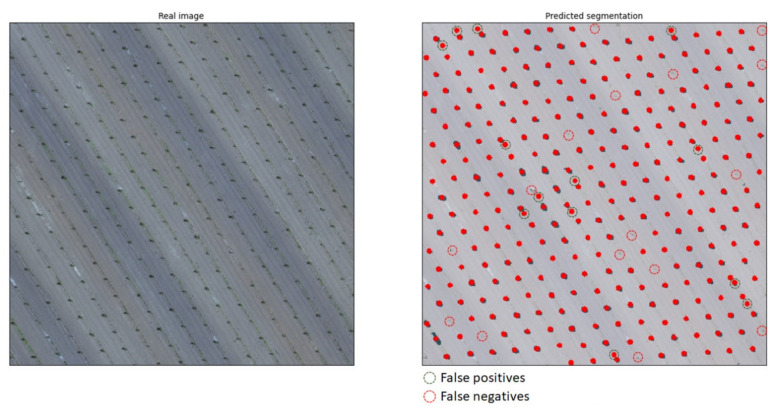
Undersampled orthomosaic of an orchard with young trees featuring small-sized canopies (**left**) and the segmentation predicted by the model (**right**).

Case B. The second use case was an undersampled orthomosaic of an orchard with young trees (Figure 9). It was observed that even though the canopies were significantly small, the trained model was able to achieve a high accuracy of 90.5% with only 5.3% FNs and 4.3% FPs.

**Figure 10:**
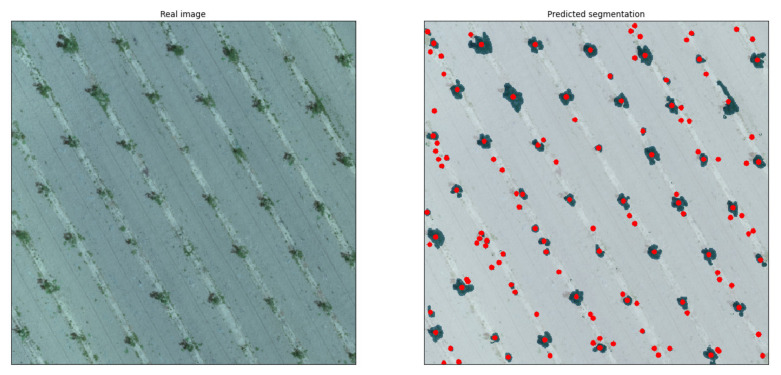
Undersampled orthomosaic of an orchard with small canopies, not treated for weeds (**left**), and the segmentation predicted by the model (**right**).

Case C. Finally, an orthomosaic with a higher resolution compared to the previous case of an orchard with small-sized canopies was undersampled and tested. However, the presence of developed weeds dispersed throughout the orchard produced many FPs in the segmentation, as seen in Figure 10.

**Figure 11:**
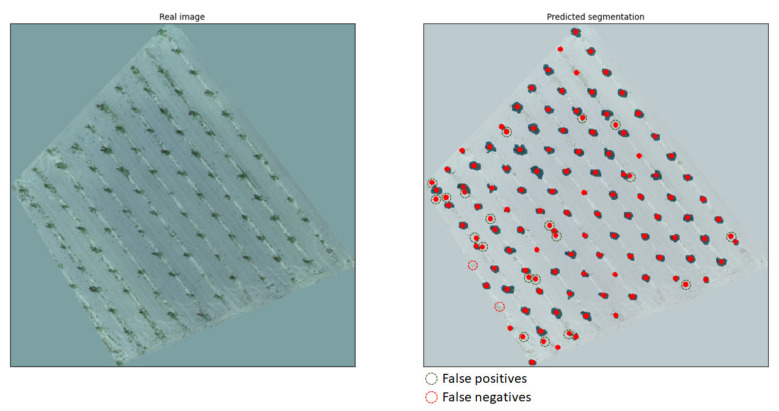
Complete orthomosaic of a study orchard with trees with small-sized canopies, not treated for weeds (**left**), and the segmentation predicted by the model (**right**).

Even though a rule-based condition could eliminate such small segmentations, this could be counterproductive for cases with young-aged trees with small canopies. However, the original orthomosaic, as seen in Figure 11, produced significantly fewer FPs compared to the undersampled one above.

The accuracy achieved for the orthomosaic was notably high, reaching 82%, and the segmentation prediction showed 16.4% FPs and only 1.6% FNs. It is worth mentioning that all the FPs were recognized as trees due to the presence of large surfaces covered by weeds, simulating the size and the shape of the top view of the tree canopy. This indicates that the model can be expected to demonstrate excellent performance with weed-free orchards. Furthermore, the FNs were located at the edges of the orthomosaic where part of the canopy of the respective trees was missing.

### 4.3. Ablation Study

The aim of this section is to demonstrate the effectiveness of the additional components (layers) and modifications that were added to the U-net architecture. The scope of evaluation in this paragraph is the effectiveness of the dropout layer that was placed between the two convolutional layers in each step. Even though a dropout layer makes intuitive sense since it improves generalization by mitigating overfitting, its actual effect should be investigated. A baseline (vanilla) U-net with no dropout layer included and a variant with the dropout layer placed before the max-pooling and the up-convolution layers were tested in comparison to the proposed variant. All models were trained with the RGB images dataset, since it was selected as the best method, and the results of the three trained models, including the proposed one, are presented in Table 6.

An approach without dropout overfitted the model and this was evident because the training accuracy was high while the validation and testing accuracies were low. This poor performance was also reflected in the number of epochs required for the model to achieve proper training, which was significantly lower (approximately 10–20 epochs) than the other approaches. Adding a dropout layer allows the model to train for a longer time (<40 epochs); however, the position of the dropout layer affects the performance of the model [37]. The variant where the dropout layer preceded the max-pooling layer performed measurably worse since, in a general sense, both dropout and pooling layers reduce learned information. The ideal combination arises when the dropout layer is placed between the two convolutional layers, since the network maintains a balance between learning and forgetting information from the input images.

### 4.4. Comparison with Baselines and Other Methods

A comparison of the proposed approach with other traditional computer vision techniques, unsupervised machine learning methods, object detection approaches, and other image segmentation deep learning techniques is presented in this section. For all methods, baseline versions were used with minor tuning of parameters. For the traditional computer vision techniques, blob, feature, and color detection were implemented with the assistance of OpenCV Python library [38]. Specifically, for the feature detection, oriented FAST and rotated BRIEF (ORB) was used as a baseline. With regard to the unsupervised machine learning approach, a K-means algorithm [39] was implemented from Python’s SciKit-Learn library [40]. For the object detection approach, the single shot detection (SSD) algorithm [41] with a ResNet50 [42] backbone was used, and for the segmentation approach, the Mask R-CNN algorithm with a ResNet101 [42] backbone, both implemented with the Keras library [43] with the Tensorflow backend [44]. Since all methods have different ways to extract information from images, the characterization of FPs and FNs was conducted by a domain expert agronomist. The total percentage of both FP and FN instances was used as a metric of comparison, and all methods were tested on the same test images from the study. The supervised learning algorithms were trained with the default parameters and with early stopping on the same training dataset. The results for all methods are presented in Table 7.

Blob detection performed poorly on use cases 1 and 2 due to the canopies being brown or leafless, on 4 and 5 due to the canopies’ shadows, and on 7 due to the matching green color on the weed-rich ground. On use case 3, no significant drawbacks were noted. Feature detection resulted in too many FP identifications in all cases because of the leaf-like appearances of most objects present in the aerial orchard photos. Color detection achieved better performance on use cases 3–6 compared to the previous two methods, but with manual tweaking of the color values for each image separately; however, when foliage and ground color bore a resemblance, there were almost no identifications. When K-means was tuned to create two clusters, for trees and backgrounds, it took into account all pixels that belonged to weeds or similar fauna. The algorithm trained with SSD was able to find most trees; however, the locations of the tree trunks, which were computed as the center of the bounding box, had noticeable deviations from the ground truth. Finally, Mask R-CNN is a two-stage approach but, even though it performed similarly to the proposed U-net approach, the generated model was five to ten times larger (the size of the proposed U-net-based model was ~22 Mb), thus rendering the lightweight implementation prerequisite as null. All methods offer benefits and drawbacks; however, it is evident that, to meet all requirements needed to tackle the problem at hand, the proposed U-net approach appears to be the optimal one.

## 5. Discussion

The present study is an initial attempt to address the problem of accurately mapping orchards via UAS. The primary focus was to construct a methodology of tree segmentation and mapping of orchards. During the testing phase of the models, useful insights were produced, along with some outcomes that showed both FP and FN misidentifications. In general, the FPs in the presented system referred to:

- Identification of shrubs and weeds as tree canopies; and
- Segmentation splits of a single instance into multiple high-density instances.

On the other hand, the FNs referred to:

- Circumstantial inadequacy in identifying small canopies; and
- Limitations in identifying trees with leafless canopies.

Considering the preprocessing method that was used, more outcomes can be discussed. For example, the simple EQ, according to the original image brightness and the size of the trees, either produced FPs next to canopies, most of them being weeds, or failed to find the trees entirely, especially if their canopy was small in size. The CLAHE methodology, a valuable tool performing well under different brightness conditions, slimmed down the canopies to a higher degree than desirable, leading to different shapes and sizes compared to the actual canopy. In many cases, this slimming splits canopies in two, which meant that the size of the tree and the location of its trunk could be incorrectly calculated. When the images were transformed into the HSV colorspace, the trained model performed well in identifying rough shapes, yet missed some obvious canopies which were not missed by other methods, leading to a high number of FNs. The fused approach demonstrated that the shortcomings of each method affected the predicted segmentations, therefore leading to models with worse performance than their best-performing counterparts. Nevertheless, the RGB model achieved the highest training and validation accuracy, the best testing accuracy, and the best performance considering FPs and FNs. This approach demonstrated robustness with all types of orchards and all seasons and for all different sizes, proving that it was the best approach for the problem at hand. Another factor that mostly affected the presence of FNs was the reshaping that images underwent in order to be fed into the training algorithm and consequently to the trained model. Resizing can compress information and in some cases this compression made small canopies “disappear”. However, even though some vital information could have been lost due to resizing, the FN errors remained at a low ratio.

The present study also demonstrated that the majority of FP segmentations were either (a) trees or bushes that were outside of the orchard, (b) developed weeds dispersed throughout the field area, or (c) split canopies resulting in two separate masks. The first category is easy to handle since the coordinates of the orchard are known and therefore any masks outside of it can be disregarded. Since the tree trunks can be calculated based on the shape of the canopy, their distances can be measured and a set of rules applied to the orchard’s structure could identify such misidentifications. The latter could serve as a good solution to address the misidentification problems caused by weeds. The third category can also be addressed by applying methods that identify the lines on which each tree is planted, therefore deducting whether the calculated coordinates of a trunk fall within an acceptable limit. All the above indicate future research directions for the continuation of this work.

The second misidentification factor can also be addressed by changing the resolution of the processed images. According to the results of the model performance evaluation on orthomosaics, in orchards with young trees featuring small canopies and filled with developed weeds, the performance was rather poor. This was attributed to the fact that the top view of the weeds was similarly colored, shaped, and sized as the very small trees within the image. This led to the identification of a large number of FPs. The resolution of the images used in the procedure played an important role in the accuracy. Running the same model on the complete orthomosaic, the results were remarkably improved, reaching 82% accuracy. This was attributed to the fact that the lower pixel resolution resulted in smoothing of the image, merging the pixels that included small weeds with the surroundings, thus making the trees stand out in the image.

Higher accuracy with regard to the overlapping area of pixels may be desired as this is a confident performance metric for model training. However, since the annotation was conducted with high detail on the canopy while the prediction was not required to outline fine details, the metric based on FP and FN predictions was additionally used to identify which method achieved the best results. Regarding the accuracy metric, the best model achieved 91% for training, 90% for validation, and 87% for testing accuracy. Considering the false predictions ratio, 13.3% was achieved for both positive and negative misidentifications of segmented canopies.

In general, image segmentation has been used in many areas; however, this is the first time, based on the authors’ knowledge, that it has been applied to UAV images of orchards. Image segmentation was selected over object detection due to a number of benefits, some of which can be summarized in the following bullet points:

- The trees’ canopy size can be distinguished;
- The trees’ canopy shape can be identified;
- Gaps in the planting scheme due to missing or defoliated and diseased trees can be identified;
- The 2D surface of the imaged canopies can be computed;
- The 3D surface and volume of the trees’ canopy can be computed;
- The trees’ ages can be approximated;
- The amount of pesticide/water needed for individual trees can be reduced by assigning proportionate amounts;
- The orchard’s yield potential can be calculated based on UAV imagery.

There are diverse possibilities for applying image segmentation to orchards and it can cover multiple aspects of operational activities in agriculture. This can be achieved with the use of deep learning, as it has proven its use in multiple occasions [45]. Additionally, semantic segmentation is an active domain with novel approaches being proposed systematically [46], some of which have direct associations with the specific shortcomings of remote sensing [47].

For the present study, U-net was utilized and tweaked to match the addressed problem and the available dataset. U-net might be considered as a relatively basic neural network considering the existence of autoencoders; however, several benefits of its use are apparent from this study:

- It achieved consistent performance >85% with all image datasets even if they had not been enhanced;
- High performance could be obtained even with a small number (~100) of images and even without image augmentation;
- The trained model could produce masks instantaneously.

These outcomes render the selection of U-net as optimal for free field deployment on UAV images. The lightness of the architecture leads to trained models which can run with on-board devices using low-power processors. This ease of application, combined with the high performance for the selected RGB model and the fact that this performance was achieved with a small dataset, leads to the conclusion that the proposed methodology is a promising start in the development of a highly sophisticated system that can identify trees in orchards and extrapolate a multitude of information useful for a variety of related operations.

The current study could be further advanced by investigating the use of other sensing tools with different capabilities and functions. These sensors might include hyperspectral or multispectral cameras, stereo/depth cameras, or thermal cameras. Each of these sensing tools has different pros and cons:

- Hyper/multispectral and thermal cameras. These cameras have multiple applications in agriculture, especially for crop monitoring. The main advantage is the high-value data related to crop and soil status. The disadvantages of this type of camera are the high computational cost that is required to transform the raw data, the high purchase cost, and the operational constraints due to various calibrations that have to take place before each flight and their dependence on weather conditions since cloud coverage greatly affects their measurements.
- Stereo/depth cameras. These are a type of camera commonly used in UGV applications due to their accurate depth perception in tandem with RGB depiction. There are two major disadvantages that constrain the use of these sensors; their low range of operational distance (most cameras have a 20 m range) and increased onboard computational requirements.
- Thermal cameras. These cameras provide high-value data, similar to the hyper- and multispectral cameras. However, they have high computational and operational costs.

However, using one of these sensors, or a combination of them, would increase the complexity of the system, adding computational costs. Since our goal was to develop a widely acceptable rapid system for on-the-go applications, we based the methodology on using RGB camera, thus making it accessible to the majority of UAS users. In this study, an initial approach for developing a simple tree segmentation system that provides instant and accurate results was proposed. Evaluating the use of the abovementioned sensors is part of our future plans for further development.

The proposed system can serve as a tool for identifying the locations of trees and obstacles within orchards and can be used as part of situation awareness and path planning for agricultural robots and autonomous vehicles. In future work, this model could serve as a UAV-based scouting tool in a UAV–UGV synergetic scheme for autonomous UGV operations within orchards. Additionally, this system can identify gaps within tree rows, thus serving as a subsystem of a farm management information system (FMIS).

## 6. Conclusions

This study addressed the problem of accurately identifying and segmenting tree canopies in a variety of orchards from UAS-captured images. The potential uses of tree segmentation cover a variety of applications, such as, for example, mapping orchard environments in order to identify the coordinates of tree trunks for autonomous ground vehicle navigation. Additionally, the system can serve as a tool to properly calculate the volume of tree canopies within orchards and consequently estimate the trees’ ages and yield potential. These operations are crucial for the next age of precision agriculture, in which on-field visual inspection by experts will be less frequent, or extensive and less time-consuming. Agricultural environments are highly complex; therefore, the ability to accurately segment tree canopies, regardless of the growth stage and the season, provides added value to any subsequent operations that take place within orchards.

The proposed approach employed a deep learning architecture, namely U-net, to create a model able to segment tree canopies from UAS-captured images. The implemented dataset was composed of images from three different orchards at different seasons throughout the year, growing trees of different ages and with different canopy sizes. The achieved variability of the dataset resulted in obtaining images that fell under seven different use cases. The best-trained model achieved 91%, 90%, and 87% accuracy for training, validation, and testing, respectively. The results of the test dataset were also hand-examined by experts in order to identify false positive and false negative instances of the produced segmentation. The mean of all false positive instances throughout the whole test dataset was 7.49% and for all false negative instances it was 5.81%. The trained model was also tested on never-before-seen orthomosaic images or orchards based on two methods in order to tackle issues with out-of-the-field boundary transparent pixels in the image. Even though the trained model did not contain orthomosaic images, it achieved performance levels that reached up to 99%, demonstrating the robustness of the proposed approach. Additionally, this study revealed issues that are present in computer vision tasks in highly complex environments, such as in agricultural production. These issues have been documented and will be the focus of upcoming studies. Other future plans include the verification of the present study’s results by testing and evaluating the performance of the trained models on different types of trees and orchard structures. Additionally, auxiliary methodologies will be developed to address the problem of densely located or merged false positives.

## Figures and Tables

**Table 1:** Orchards’ characteristics and categorization into separate use cases.

Use Case No.	Yearly Season	Weeds Coverage	Canopy Size	Foliage Color	Ground Color
1	Autumn	Low	-	Brown	Brown
2	Autumn	Low	-	Mixed	Brown
3	Summer	Low	Small	Green	Brown
4	Summer	Low	Medium	Green	Brown
5	Summer	Low	Medium	Green	Mixed
6	Summer	Low	Large	Green	Brown
7	Summer	High	Large	Green	Green

**Table 2:** Information concerning the flights performed in each use case for the acquisition of images and the creation of the orthomosaics used in the study.

Use Case No.	Acquisition Date	Number of Trees	Number of Images	Overlap	GSD	Air Speed (m/s)	Cloud Coverage (%)
1	1 November 2018	1399	283	75%	1.3	<3	49
2	30 August 2020	569	522	75%	1.3	3	32
3	19 June 2020	358	330	75%	1.3	<3	5
4	3 June 2020	506	244	75%	1.5	<3	35
5	12 August 2020	2118	510	75%	1.5	<3	40
6	7 May 2019	296	193	75%	1.3	<3	12
7	15 May 2020	632	465	75%	1.3	<3	5

**Table 3:** Accuracy (dice coefficient) for investigated methods of segmentation.

Image Colorspace	RGB	EQ	CLAHE	HSV	RGB + HSV	CLAHE + HSV
Channels	3				6	
Training accuracy	0.91	0.90	0.90	0.92	0.91	0.91
Validation accuracy	0.90	0.88	0.89	0.90	0.89	0.90
Testing accuracy	0.87	0.77	0.86	0.86	0.85	0.86

**Table 4:** Overall performance evaluation, expressed as percentages (%), of the models examined in the test set of the study, in terms of false positives (FPs), false negatives (FNs), and their sum ratios over the total number of trees in the test set.

Image Colorspace	RGB	EQ	CLAHE	HSV	RGB + HSV	CLAHE + HSV
FPs (%)	7.49	9.41	16.17	7.57	7.49	4.99
FNs (%)	5.81	8.73	15.17	6.48	10.66	16.22
Total misidentifications (%)	13.30	18.14	31.34	14.05	18.16	21.21

**Table 5:** Performance evaluation of the RGB model (best performing) applied to the separate test images for each use case, expressed as percentages (%) of false positives, false negatives, and their sum total.

Test Image	1	2	3	4	5	6	7	8	9	10	Mean
Use Case	2	1	5	4	4	6	6	5	7	3	
FPs (%)	7.69	8.33	16.67	9.09	2.08	1.82	2.33	12.64	14.29	0.00	7.49
FNs (%)	0.00	4.17	4.17	18.18	0.00	1.82	0.00	3.45	2.38	23.94	5.81
Total (%)	7.69	12.50	20.83	27.27	2.08	3.64	2.33	16.09	16.67	23.94	13.30

**Table 6:** Results of the ablation study for the baseline (vanilla) U-net, a variant with a dropout layer placed after the convolutional layers, and the proposed variant.

	Vanilla	Variant No. 1	Proposed Variant
Training accuracy	0.89	0.86	0.91
Validation accuracy	0.78	0.85	0.90
Testing accuracy	0.74	0.81	0.87
FPs (%)	13.56	9.93	7.49
FNs (%)	12.48	8.74	5.81
Total misidentifications (%)	26.04	18.67	13.30

**Table 7:** Comparison of the proposed approach (in bold) with other computer vision baselines and machine learning methods using total percentage of misidentifications as a metric (sum of false positives and false negatives).

Test Image	1	2	3	4	5	6	7	8	9	10	Mean
Use Case	2	1	5	4	4	6	6	5	7	3	
Blob detection	63.65	56.81	34.35	34.57	31.73	28.00	25.75	28.54	65.39	39.57	40.84
Feature detection (ORB)	65.68	59.56	49.72	46.85	48.38	50.24	47.90	48.81	63.40	43.74	52.43
Color detection	53.88	52.96	35.32	32.27	31.12	29.62	29.03	27.51	55.88	27.45	37.50
Clustering (K-means)	52.25	54.17	40.19	39.12	38.69	36.47	36.16	36.20	53.55	42.97	42.98
Object detection (SSD)	12.34	15.28	21.68	29.16	5.92	7.03	7.05	19.39	21.01	27.10	16.60
Mask R-CNN	8.31	13.01	19.80	27.21	3.45	3.98	2.80	16.59	17.98	23.00	13.61
**Proposed U-net**	**7.69**	**12.50**	**20.83**	**27.27**	**2.08**	**3.64**	**2.33**	**16.09**	**16.67**	**23.94**	**13.30**

## Appendix A

**Table A1:** Sample images of the seven use cases included in the study.

Use Case No.	Conditions	Sample Image
1	Yearly season: Autumn Weeds coverage: Low Canopy size: - Foliage color: Brown Ground color: Brown	
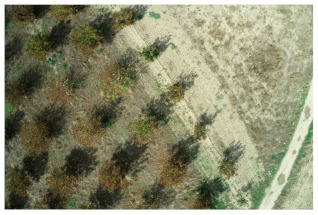
2	Yearly season: Autumn Weeds coverage: Low Canopy size: - Foliage color: Mixed Ground color: Brown	
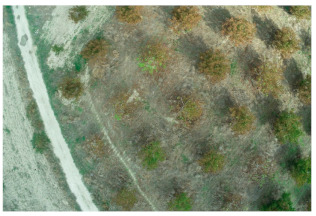
3	Yearly season: Summer Weeds coverage: Low Canopy size: Small Foliage color: Green Ground color: Brown	
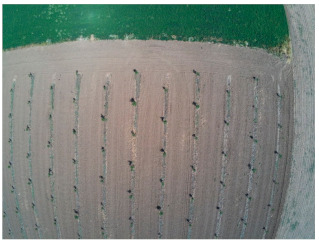
4	Yearly season: Summer Weeds coverage: Low Canopy size: Medium Foliage color: Green Ground color: Brown	
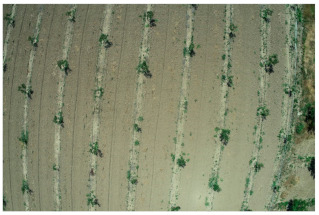
5	Yearly season: Summer Weeds coverage: Low Canopy size: Medium Foliage color: Green Ground color: Mixed	
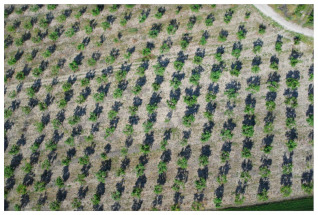
6	Yearly season: Summer Weeds coverage: Low Canopy size: Large Foliage color: Green Ground color: Brown	
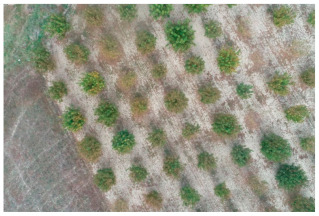
7	Yearly season: Summer Weeds coverage: High Canopy size: Large Foliage color: Green Ground color: Green	
